# Artificial Intelligence Models for Pediatric Lung Sound Analysis: Systematic Review and Meta-Analysis

**DOI:** 10.2196/66491

**Published:** 2025-04-18

**Authors:** Ji Soo Park, Sa-Yoon Park, Jae Won Moon, Kwangsoo Kim, Dong In Suh

**Affiliations:** 1 Department of Pediatrics Seoul National University College of Medicine Seoul Republic of Korea; 2 The Institute of Convergence Medicine with Innovative Technology Seoul National University Hospital Seoul Republic of Korea; 3 Department of Physiology, College of Korean Medicine Wonkwang University Iksan Republic of Korea; 4 Department of Medicine Seoul National University College of Medicine Seoul Republic of Korea

**Keywords:** machine learning, respiratory disease classification, wheeze detection, auscultation, mel-spectrogram, abnormal lung sound detection, artificial intelligence, pediatric, lung sound analysis, systematic review, asthma, pneumonia, children, morbidity, mortality, diagnostic, respiratory pathology

## Abstract

**Background:**

Pediatric respiratory diseases, including asthma and pneumonia, are major causes of morbidity and mortality in children. Auscultation of lung sounds is a key diagnostic tool but is prone to subjective variability. The integration of artificial intelligence (AI) and machine learning (ML) with electronic stethoscopes offers a promising approach for automated and objective lung sound.

**Objective:**

This systematic review and meta-analysis assess the performance of ML models in pediatric lung sound analysis. The study evaluates the methodologies, model performance, and database characteristics while identifying limitations and future directions for clinical implementation.

**Methods:**

A systematic search was conducted in Medline via PubMed, Embase, Web of Science, OVID, and IEEE Xplore for studies published between January 1, 1990, and December 16, 2024. Inclusion criteria are as follows: studies developing ML models for pediatric lung sound classification with a defined database, physician-labeled reference standard, and reported performance metrics. Exclusion criteria are as follows: studies focusing on adults, cardiac auscultation, validation of existing models, or lacking performance metrics. Risk of bias was assessed using a modified Quality Assessment of Diagnostic Accuracy Studies (version 2) framework. Data were extracted on study design, dataset, ML methods, feature extraction, and classification tasks. Bivariate meta-analysis was performed for binary classification tasks, including wheezing and abnormal lung sound detection.

**Results:**

A total of 41 studies met the inclusion criteria. The most common classification task was binary detection of abnormal lung sounds, particularly wheezing. Pooled sensitivity and specificity for wheeze detection were 0.902 (95% CI 0.726-0.970) and 0.955 (95% CI 0.762-0.993), respectively. For abnormal lung sound detection, pooled sensitivity was 0.907 (95% CI 0.816-0.956) and specificity 0.877 (95% CI 0.813-0.921). The most frequently used feature extraction methods were Mel-spectrogram, Mel-frequency cepstral coefficients, and short-time Fourier transform. Convolutional neural networks were the predominant ML model, often combined with recurrent neural networks or residual network architectures. However, high heterogeneity in dataset size, annotation methods, and evaluation criteria were observed. Most studies relied on small, single-center datasets, limiting generalizability.

**Conclusions:**

ML models show high accuracy in pediatric lung sound analysis, but face limitations due to dataset heterogeneity, lack of standard guidelines, and limited external validation. Future research should focus on standardized protocols and the development of large-scale, multicenter datasets to improve model robustness and clinical implementation.

## Introduction

Accurate and timely diagnosis is essential for the treatment of pediatric respiratory illnesses, which remain a leading cause of morbidity and mortality among children worldwide [1,2]. Auscultation of lung sounds is the most widely used method of respiratory diagnosis due to its simplicity, cost-effectiveness, and safety. However, conventional auscultation requires an in-person encounter, is prone to subjective interpretation, and cannot be shared or reviewed among clinicians, leading to high interobserver variability [3].

In recent years, the development of electronic stethoscopes has enabled the digital storage and computational analysis of lung sounds, leading to the creation of large-scale databases [4,5]. Artificial intelligence (AI)–driven lung sound analysis based on these databases has opened new opportunities to enhance the accuracy and reliability of respiratory disease diagnosis [6]. Automated AI models for lung sound analysis can facilitate prompt diagnosis and monitor disease progression is particularly useful in remote areas lacking experienced pediatricians or during public health crises, such as the COVID-19 pandemic, when large-scale respiratory screenings are needed [7,8].

**Figure 1:**
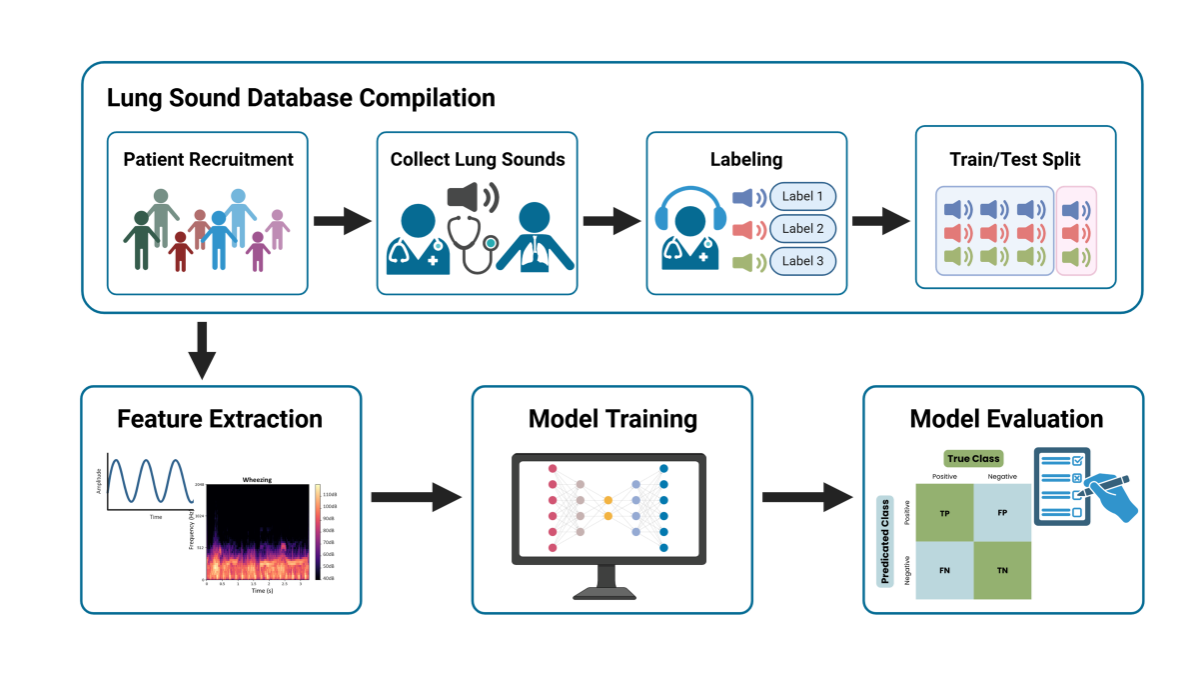
General process of developing a machine learning model for pediatric lung sound assessment.

The typical process for developing an AI model to assess pediatric lung sounds includes the following steps: (1) patient recruitment, (2) recording of lung sounds, (3) physician labeling of lung sounds, (4) database creation with separate training and testing sets, (5) feature extraction, (6) machine learning (ML) model development and training, and (7) evaluation of the model with appropriate performance metrics (Figure 1). These steps could vary between studies, as there are no standardized protocols or guidelines in this area. The current body of research demonstrates high variability in study design, dataset sizes, tasks implemented, and reported outcomes, making it difficult to draw definitive conclusions about the effectiveness of these technologies. Therefore, a comprehensive review of the existing evidence for the application of ML models in pediatric lung sound analysis is necessary.

This systematic review and meta-analysis aim to systematically assess the accuracy of reported ML models on pediatric lung sound analysis, by examining the tasks, methodologies, databases, and evaluation metrics used in original studies. Ultimately, we seek to establish the robustness of current ML models and provide insights for further research.

RQ1: Is it feasible to reliably classify pediatric lung sounds using ML models?

RQ2: How accurately can ML models classify lung sounds into different types of lung sounds or lung pathologies?

## Methods

### Search Strategy

The literature search was conducted according to the Preferred Reporting Items for Systematic Reviews and Meta-Analysis for Diagnostic Test Accuracy Studies (PRISMA-DTA) statement [9]. We followed the PRIMSA-DTA checklist (Multimedia Appendix 1 [9]). The main research question of this systematic literature review was to find studies that developed ML models for classifying abnormal lung sounds or pediatric lung pathologies using pediatric lung sound data (Textbox 1).

**Population, Intervention, Comparator, Outcome, and Study design (PICOS) for the systematic review.**

**Inclusion criteria**

Population:

- Pediatric population (age≤18 years).
- Lung sound database specified.
- Manuscript in English.

Intervention:

- Machine learning model classifying at least one type of abnormal lung sounds (wheeze, crackle, stridor, or rhonchi) or lung pathologies (pneumonia, asthma, bronchiolitis, etc).

Comparator:

- Labeling provided by the database.

Outcomes:

- At least one performance metric reported: specificity, sensitivity, accuracy, *F*_1_-score, or other specified scoring system.

Study design:

- Original studies on machine learning model development.

**Exclusion criteria**

Population:

- Adult only or adult majority.
- Database not mentioned.
- Studies focused on cardiac auscultation.

Intervention:

- No machine learning model used.

Comparator:

- No labeling provided.

Outcomes:

- No performance metric provided.

Study design:

- Review articles and validation studies of existing models.

### Database Search

We searched Medline via PubMed, Embase, Ovid, Web of Science, and IEEE Xplore using search queries including the following keywords and synonyms such as “infant,” “child, preschool,” “child,” “adolescent,” “pediatrics,” “respiratory sounds,” “auscultation,” “adventitious sound,” “wheeze,” “crackle,” “rale,” “stridor,” “rhonchus,” “machine learning,” “neural network,” and “artificial intelligence.” Exact queries are detailed in Multimedia Appendix 2. The search covered articles from January 1, 1990, to December 16, 2024.

### Eligibility Criteria

Studies were included if they were based on a pediatric population (age ≤18 years), or if more than half of the population were of pediatric age. Lung sound databases—whether public or private—needed to be specified. Only manuscripts in English were considered. Eligible studies applied original ML models for classifying at least one type of abnormal lung sound (wheeze, crackle, stridor, or rhonchi) or lung pathology (eg, pneumonia, asthma, bronchiolitis, etc). Studies without ML algorithms and studies that only performed validation of previously developed models were excluded. Labeling methods needed to be specified for comparators, and at least one performance metric was needed. Abstracts, conference proceedings, and journal papers were included.

### Study Selection

Two researchers (JSP and SYP) independently performed abstract and full-text screening. Disagreements were resolved by discussion and mediation by a third researcher (DIS). Articles in languages other than English, duplicate articles from multiple databases, and studies that did not meet the eligibility criteria for population and intervention were excluded during abstract screening. In the full-text review, duplicate studies (eg, conference abstracts later published as full journal papers) were excluded and studies not meeting eligibility criteria due to study design were excluded. EndNote 21 (Clarivate Analytics) was used during this process.

### Data Extraction

Data extraction was performed by 2 researchers (JSP and SYP) using a predefined data extraction form in Excel. Article type (conference proceeding or journal paper), first author, year of publication, and journal or conference name were extracted from Endnote. Country of data collection, age of study population, database characteristics, recording device, sample size, train-test split, classification task (eg, wheeze detection, multiclass classification of lung sounds, and asthma classification), feature extraction methods, summary of ML models, and performance metrics were collected. For binary classification, confusion matrix data—true positive (TP), true negative (TN), false positive (FP), and false negative (FN)—were extracted, and if not provided, they were calculated from the sample size and performance metrics.

### Meta-Analysis

We conducted meta-analyses for binary classification tasks: wheeze detection and abnormal lung sound detection. Sensitivity and specificity were calculated based on the TP, TN, FP, and FN extracted from the included studies. Sensitivity was defined as the proportion of correctly identified abnormal lung sounds (TP/[TP + FN]), and specificity as the proportion of correctly identified normal lung sounds (TN/[TN + FP]). A pooled analysis of sensitivity and specificity and their CI was conducted with a bivariate metanalysis using a random-effects model [10]. The heterogeneity among the studies was established by the Zhou and Dendukuri approach, with inconsistency values (I2) greater than 50% being considered as moderate heterogeneity and values above 75% indicating high heterogeneity. Meta-analysis was conducted using the mada package in R software (version 4.3.2; R Foundation for Statistical Computing).

### Quality Assessment

Researchers assessed the quality of the study based on the revised tool for the Quality Assessment of Diagnostic Accuracy Studies (QUADAS-2) [11]. Study quality was assessed based on 2 factors: risk of bias and applicability. Risk of bias was high if the systematic limitation in the study design or conduct was likely to influence the results. Applicability referred to the extent to which the study population, index test, or reference standard was representative of the review question. Risk of bias was evaluated in 4 domains: patient selection, index test, reference standard, and flow and timing. Applicability was assessed in 3 domains: patient selection, index test, and reference standard. The QUADAS-2 was modified to suit ML-based diagnostic studies [12,13] (Table 1).

**Table 1:** Domain details for risk of bias and applicability.

Domains		Signaling question	Review question
**Risk of bias domains**			
	Patient selection	Was a consecutive or random sample of patients or lung sounds enrolled in the test set? Was a case-control design avoided? Did the study avoid inappropriate exclusions?	—^a^
	Index test	Were the index test results independent of the patient characteristics that may affect the reference standard? Was the performance metric evaluated in a prespecified independent test set?	—
	Reference standard	Is the reference standard (physician labeling) likely to correctly classify the target condition? Were the reference standard (physician labeling) labeled without knowledge of the results of the index test?	—
	Flow and timing	Was there an appropriate interval between the index test and reference standard? Did all the patients receive the same reference standard?	—
**Applicability domains**			
	Patient selection	—	Pediatric patients that need diagnosis of respiratory conditions.
	Index test	—	Machine learning model with lung sounds as input and type of lung sound or lung pathology as output
	Reference standard	—	Labeling of physicians

^a^Not applicable.

## Results

**Figure 2:**
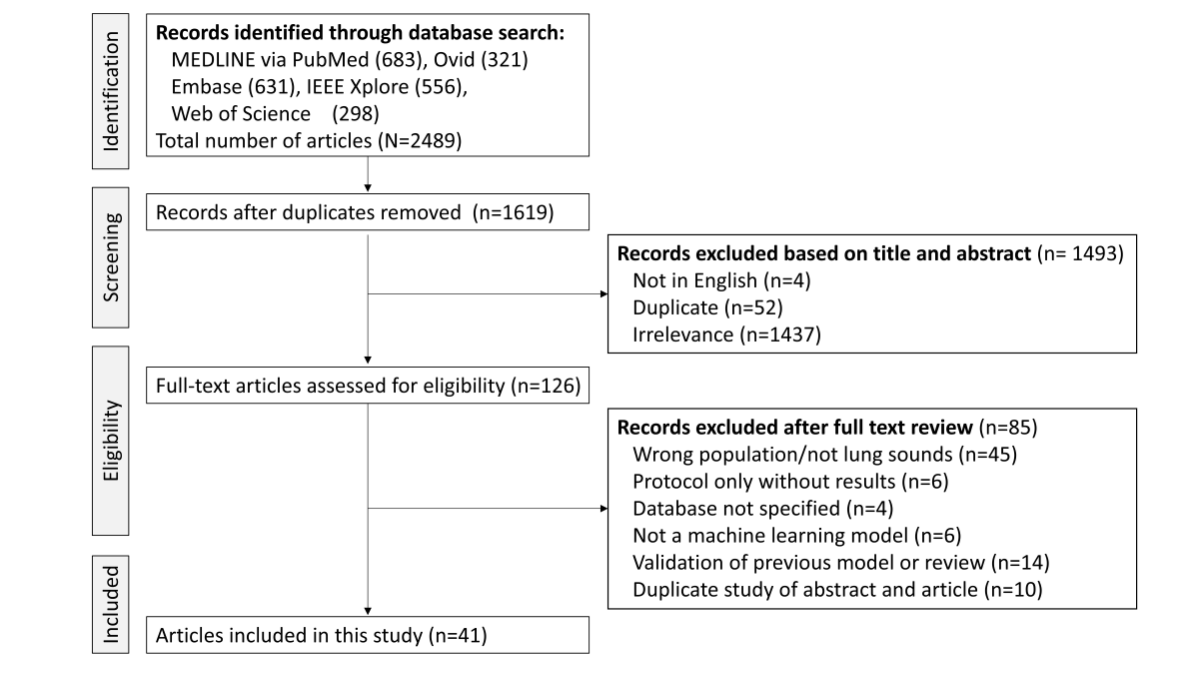
Preferred Reporting Items for Systematic Reviews and Meta-Analyses (PRISMA) flow diagram.

Systematic search from the 3 databases yielded a total of 2191 articles including conference abstracts, conference proceedings, and journal papers. After removing duplicates and conducting abstract screening, 126 full-text articles were assessed for eligibility. A total of 55 studies were excluded based on criteria such as inappropriate population, incorrect input data for classifiers, missing results, or lack of original ML models. A total of 41 studies were included in the final analysis [14-54] (Figure 2).

The included studies spanned from the 1990s (2 studies), through the 2010s (12 studies), to the 2020s (27 studies). A total of 29 studies focused on classifying abnormal lung sounds; of these, 15 aimed for binary classification and 14 pursued multiple classification. In binary classification, wheeze detection was the most researched topic of the studies. Twelve studies targeted classifying the diagnosis, prognosis, or severity of specific lung pathologies, such as pneumonia and asthma. Among these, 5 studies used binary classification, and 7 studies used multiple classification approaches. Pneumonia was the most frequently studied condition, followed by asthma. One study aimed to classify the severity of cystic fibrosis (CF) based on lung sounds (Table 2).

The performance metrics reported in these studies included accuracy, sensitivity, specificity, *F*_1_-score, area under the receiver operating characteristic curve, and other unique metrics calculated using a combination of these metrics. Detailed information on the included studies—such as the databases used, sample sizes, tasks, training and test set sizes, feature extraction methods, and ML models—are presented in Multimedia Appendix 3 [14-54].

**Table 2:** Summary of included studies. Included studies are shown as reference numbers of the text.

Data type and binary/multiple classes			Task		Included studies
**Lung sound** **(n=10)**					
	Binary (n=15)	Wheeze (n=7) Abnormal (n=4) CAS^a^ (n=1) Crackle (n=1) Wheeze vs crackle (n=1) Dissimilar sounds (n=1)		[14,18-20,22,24,27,30,32,34-36,47,48,53]	
	Multiple (n=4)	Normal, wheeze, rale, stridor, etc.		[25,28,54,55]	
	Binary and multiple (n=10)	Adventitious detection, normal, wheeze, rale, stridor, etc.		[16,37-40,42,45,46,49,50]	
**Lung pathology (n=12)**					
	Binary (n=5)	Asthma (n=1) Bronchitis (n=2) Bronchiolitis (n=1) Pneumonia (n=2)		[17,21,23,33,52]	
	Multiple (n=7)	Asthma status (n=1) Asthma/croup/pneumonia (n=2) CF^b^ severity (n=1) Pneumonia/wheezing disorder/bronchiolitis (n=1) CAP^c^ progression state (n=2)		[15,26,29,31,41,43,44]	

^a^CAS: continuous adventitious sound.

^b^CF: cystic fibrosis.

^c^CAP: community-acquired pneumonia.

We extracted 2×2 confusion matrices (TP, FP, TN, and FN) for binary classification studies. Of the 7 studies that examined wheeze detection, 6 provided confusion matrix data (Table 3). For abnormal lung sound detection, 7 of the 15 studies reported confusion matrix data (Table 4).

**Figure 3:**
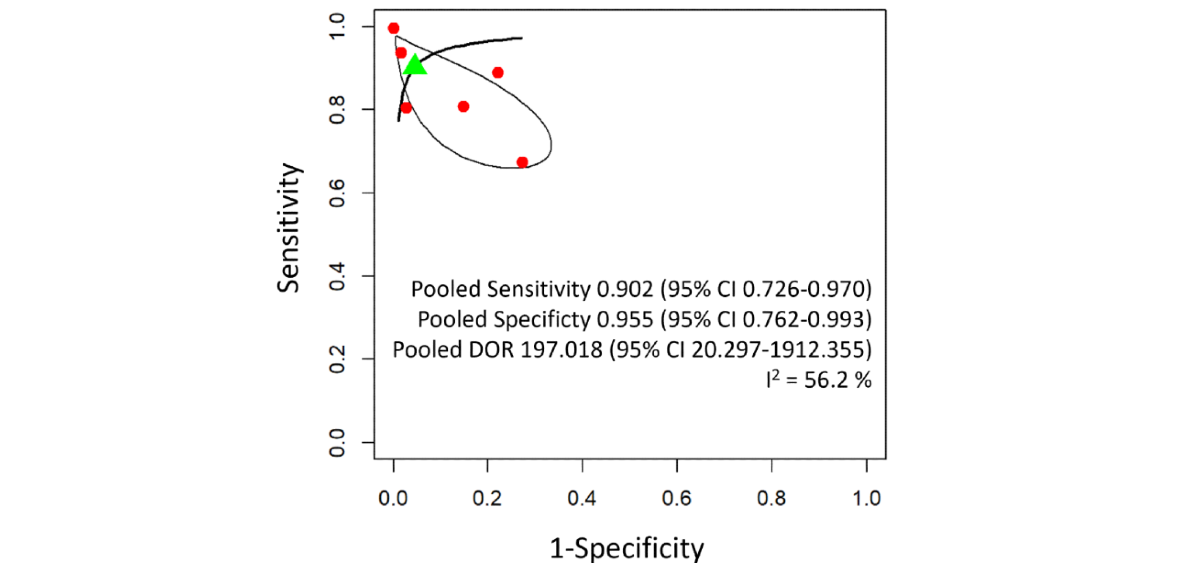
Summary receiver operating characteristic curve for wheeze detection machine learning models. The solid black line represents the estimated summary receiver operating characteristic curve. The green triangle marks the pooled sensitivity and specificity estimated from the bivariate meta-analysis, with the 95% CI ellipse around it. Red dots represent the sensitivity and specificity of individual studies. DOR: diagnostic odds ratio.

**Figure 4:**
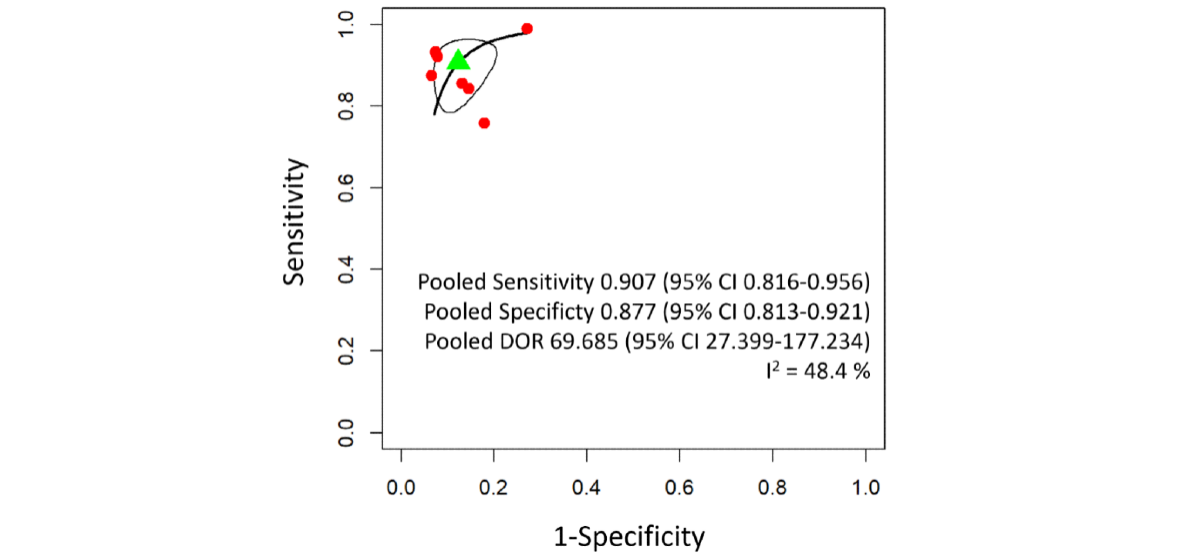
Summary receiver operating characteristic curve for abnormal lung sound detection machine learning models. The solid black line represents the estimated summary receiver operating characteristic curve. The green triangle marks the pooled sensitivity and specificity estimated from the bivariate meta-analysis, with the 95% CI ellipse around it. Red dots represent the sensitivity and specificity of individual studies. DOR: diagnostic odds ratio.

The pooled sensitivity and specificity of wheeze detection models were 0.902 (95% CI 0.726-0.970) and 0.955 (95% CI 0.762-0.993), respectively. There was moderate between-study heterogeneity (Figure 3). The pooled sensitivity of abnormal lung sound detection models was 0.907 (95% CI 0.816-0.956) and the specificity was 0.877 (95% CI 0.813-0.921). Low heterogeneity was found (Figure 4).

**Table 3:** Confusion matrix of studies on wheeze detection.

Author (year)	True positive (TP)	False positive (FP)	True negative (TN)	False negative (FN)
Forkheim et al (1995) [14]	64	38	102	31
Mazic et al (2015) [19]	268	2	2572	1
Milicevic et al (2016) [20]	N/A^a^	N/A	N/A	N/A
Kuo et al (2021) [30]	30	1	62	2
Kim et al (2022) [34]	83	5	179	20
Nguyen et al (2022) [36]	401	142	502	50
Park et al (2023) [48]	38	8	46	9

^a^Not available.

**Table 4:** Confusion matrix of studies on abnormal lung sound detection.

Author (year)	True positive (TP)	False positive (FP)	True negative (TN)	False negative (FN)
Emmanouilidou et al (2012) [16]	N/A^a^	N/A	N/A	N/A
Khan et al (2017) [21]	71	06	71	06
Emmanouilidou et al (2018) [22]	N/A	N/A	N/A	N/A
Liu et al (2019) [27]	N/A	N/A	N/A	N/A
Zhang et al (2022) [37]	295	178	822	94
Li et al (2022) [38]	310	103	1295	22
Zhang et al (2022) [39]	N/A	N/A	N/A	N/A
Babu et al (2022) [40]	N/A	N/A	N/A	N/A
Hu et al (2023) [42]	385	281	759	4
Ngo et al (2023) [45]	N/A	N/A	N/A	N/A
Ngo et al (2023) [46]	328	151	889	61
Pessoa et al (2023) [49]	N/A	N/A	N/A	N/A
TaghiBeyglou et al (2024) [50]	N/A	N/A	N/A	N/A
Chowdhury et al (2024) [51]	18	3	20	3
Crisdayanti et al (2024) [52]	4859	366	5239	690

^a^Not available.

A total of 29 distinct databases were identified across the included studies (Table 5). Eight databases labeled lung pathologies including 6 studies that labeled a single lung pathology (pneumonia in 2 studies, asthma in 2 studies, bronchitis in 1 study, and CF in 1 study) and 2 studies that labeled multiple lung pathologies. Seventeen studies labeled abnormal lung sounds as follows: 6 studies with binary labels of wheeze/normal or wheeze/nonwheeze, 3 studies with binary labels of abnormal (or adventitious)/normal, and 8 studies with multiple abnormal lung sound labels (eg, wheeze, crackle, and stridor) labels. Labeling methods or personnel were specified in 19 databases and not specified in 6. Details of each database are available in Multimedia Appendix 4 [14-29,31-37, 39-41,43-46,48-54].

The most used database was Shanghai Pediatric Respiratory Sound Database, a Chinese database collected from Shanghai University Hospital. The database was used for a challenge in the IEEE BIOCAS 2022 and again in 2023 with an additional test set [37]. This is the only open-source pediatric lung sound database available. Another public database, the International Conference on Biomedical and Health Informatics 2017 includes only a small number of pediatric participants [55]. The International Conference on Biomedical and Health Informatics 2017 database was used for pretraining of models or for external validation in some studies.

**Table 5:** Summary of databases used in reviewed studies.

Database	Size	Label type	Availability	Studies
SPRSound^a^	292 subjects	Lung sound	Public	[32,35,37,39,40,45,46,49,50,53,54]
ICBHI^b^	49 subjects	Lung sound	Public	[27,48,52,53]
Liu et al (2019)	12 recordings	Disorder	Private	[25,31]
CCAP-LSD^c^	198 subjects	Disorder	Private	[43,44]
Forkheim et al (1995)	710 patterns	Lung sound	Private	[14]
Rietveld et al (1999)	60 subjects	Disorder	Private	[15]
Emmanouilidou et al (2012)	28 recordings	Lung sound	Private	[16]
Khan et al (2012)	40 subjects	Disorder	Private	[17]
Jin et al (2014)	21 subjects	Lung sound	Private	[18]
Mazic et al (2015)	16 subjects	Lung sound	Private	[19]
Milicevic et al (2016)	863 samples	Lung sound	Private	[20]
PERCH	1157 subjects	Lung sound	Private	[21]
Khan et al (2017)	254 sounds	Lung sound	Private	[22]
Mohamed et al (2018)	48 patients	Disorder	Private	[23]
Gouda et al (2019)	446 sounds	Lung sound	Private	[24]
Grzywalski et al (2019)	50 subjects (test set)	Lung sound	Private	[25]
Liu et al (2019)	222 subjects	Lung sound	Private	[27]
Kotb et al (2020)	116 children	Lung sound	Private	[26]
Karimizadeh et al (2021)	209 subjects	Disorder	Private	[27]
Kuo et al (2021)	94 subjects	Lung sound	Private	[28]
Cheng et al (2021)	73 samples	Lung sound	Private	[32]
Gelman et al (2022)	1118 records	Disorder	Private	[33]
Kim et al (2022)	76 subjects	Lung sound	Private	[34]
Nguyen et al (2022)	1095 recordings	Lung sound	Private	[36]
DeepBreath	572 subjects	Disorder	Private	[41]
Park et al (2023)	1112 clips	Lung sound	Private	[48]
R.A.L.E.	>50 recordings	Lung sound	Public	[29]
Chowdhury et al (2024)	19 toddlers	Disorder	Private	[51]
Crisdayanti et al (2024)	675 patients	Lung sound	Private	[52]

^a^SPRSound: Shanghai Pediatric Respiratory Sound Database.

^b^ICBHI: International Conference on Biomedical and Health Informatics.

^c^CCAP-LSD: Cystic Fibrosis Center for Advanced Pediatric Learning and Study Data.

Various feature extraction methods were used (Table 6). The most frequently used method was Mel-frequency cepstral coefficients (MFCC), which was used in 11 studies. MFCC captures the overall shape of the spectral envelope in a compressed form across the Mel scale, a perceptual frequency scale that reflects human auditory sensitivity. The second most widely used method was the (log) Mel-spectrogram, which was applied in 9 studies. This technique provides a visual representation of an audio signal’s frequency content over time, with frequencies converted to the Mel scale. Other Fourier transform-based methods, including short-time Fourier transform (STFT) and spectral features derived from the Fourier spectrum, were used to analyze the frequency content of the signals. Statistical features such as kurtosis, sample entropy, and time-frequency domain characteristics were also used in some studies. Other techniques such as wavelet transformations (continuous and discrete), cochleogram, and time-varying linear predictive coding were also used to extract features from the lung sound recordings.

**Table 6:** Feature extraction methods.

Feature extraction	Features	Studies
Short-time Fourier transform (STFT) or spectrogram	FT^a^ decomposes a signal into its frequency components, assuming the frequency is stationary throughout the entire time sequence. STFT analyzes how frequencies change over time by dividing the signal into overlapping short frames and applying FT to each frame. This provides a time-frequency representation, assuming stationarity within each frame. A spectrogram is a visual representation of the STFT that displays the magnitude or power as a function of time and frequency, providing a time-frequency representation of the signal.	[14,15,21,24,25,35,36,38,49,54]
Fourier transform-based spectral feature	Derived from the Fourier spectrum of respiratory sounds, the process involves converting the audio into a time series, applying the FT, and deriving features like spectral bandwidth, centroid, roll-off, and chroma.	[33]
(log) Mel-spectrogram	A visual representation of an audio signal’s frequency content over time, where the frequencies are converted to the Mel scale, which is based on human perception of pitch. It shows how the energy of different frequency bands evolves over time. The log Mel-spectrogram is computed by taking the logarithm of the Mel-spectrogram values, which helps to compress the dynamic range and emphasize lower energies.	[23,27,34,37,41,43,44,50,52]
Mel-frequency cepstral coefficients (MFCC)	MFCC are features derived by applying a Discrete Cosine Transform to the log Mel-spectrogram, capturing key spectral characteristics of an audio signal, often used in speech and audio recognition.	[17,19,20,24,26,28,37,39,42,48,51]
Statistics	A set of features that capture time-domain, frequency-domain, and complexity aspects of breath sounds, including kurtosis, sample entropy, lung sound power ratio, respiratory rate, breathing cycle metrics, peak frequency, and wheezing characteristics, providing key insights for diagnosing respiratory diseases.	[18-20,29,30,32,39]
Continuous wavelet transformation (CWT)	Technique to analyze the frequency content of a signal over time using wavelets—small, localized functions that can stretch or compress to capture details at various scales. The CWT provides good time resolution for high-frequency components and good frequency resolution for low-frequency components, making it suitable for analyzing nonstationary signals.	[45,46]
Discrete wavelet transformation	A sampled version of the CWT that selects scales and positions based on powers of 2. The signal is passed through a series of high-pass and low-pass filters, down-sampled by a factor of 2 at each level, and decomposed into wavelet coefficients representing different frequency bands and time scales.	[24,47]
Cochleogram	A time-frequency representation of an audio signal that mimics human auditory processing. Sound signal is passed through bandpass filters based on the Equivalent Rectangular Bandwidth scale, simulating the cochlear frequency resolution, and smoothed and compressed, providing a biologically-inspired representation of the signal’s spectral content.	[22]
Time-varying linear predictive coding (TVLPC)	A feature extraction technique for nonstationary breath sounds, where the signal is modeled as a linear combination of past samples with time-varying filter coefficients. Unlike traditional LPC, TVLPC allows these coefficients to change over time by expressing them as a linear combination of basis functions, capturing the dynamic spectral characteristics essential for accurately classifying respiratory conditions.	[31]

^a^FT: Fourier Transform.

A wide range of ML models were used in the included studies for classifying lung sounds and diagnosing respiratory conditions (Table 7). Convolutional neural networks (CNNs) were the most frequently used, featured in 12 studies, in combination with other architectures like recurrent neural networks in 2 studies. Artificial neural networks were also popular, and were used in 10 studies. Residual network (ResNet), a deep CNN architecture with residual connections, was used in 6 studies. Support vector machines (SVMs), another widely used model, was used in 8 studies for optimizing decision boundaries. Other models explored included transformer, hidden Markov models, k-nearest neighbors, ensemble models, and probabilistic classifiers like Naïve Bayes. The choice of model depended on factors such as the nature of the data, the complexity of the classification task, and desired performance metrics.

**Table 7:** Summary of the machine learning models used by studies in this review.

Model name	Features	Studies
Artificial neural network	Comprised of interconnected nodes arranged in layers. Data flows from the input layer, through hidden layers, to the output layer. Nodes are connected by weighted links that are tuned during training.	[14-16,24-27,29,30,33,44]
Convolutional neural network (CNN)	A type of neural network optimized for processing grid-like data such as images. It uses convolutional layers to extract spatial features and pooling layers to reduce dimensionality.	[23,27,34-36,40,41,47,49,51,52,54]
Residual Network (ResNet)	A deep CNN architecture that introduces residual connections, which skip one or more layers and alleviate the vanishing gradient problem. This enables the training of very deep networks.	[35,39,40,42,43,47]
Support vector machine (SVM)	A discriminative classifier that constructs a hyperplane or set of hyperplanes in a high-dimensional space to maximize the margin between classes. It aims to find the optimal decision boundary.	[15,17-22,32,48]
CNN + recurrent neural networks (RNNs)	A hybrid architecture that combines the spatial feature extraction capabilities of CNNs with the temporal modeling abilities of RNNs. This allows for capturing both spatial and sequential patterns in data.	[43,44]
Hidden Markov model	A probabilistic model that assumes the system being modeled is a Markov process with hidden states. It consists of a sequence of state variables and observed variables, with transitions between states governed by probability distributions.	[28,31]
K-nearest neighbors (KNN)	A nonparametric method that classifies data points based on the majority class among their K nearest neighbors in the feature space. It assigns unlabeled examples to the class most common among the K closest labeled instances.	[21]
Two-level ensemble model	A hierarchical ensemble architecture with 2 levels of model combination. Level 1 separates models by gender to capture data heterogeneity. It trains specialized models for each gender group. Level 2 trains multiple diverse models within each gender group using AutoGluon, an AutoML framework. These multiple models are strategically generated and combined to solve a computational intelligence problem.	[39]
Naïve Bayes	A probabilistic classifier based on applying Bayes’ theorem with strong independence assumptions between features. It calculates the probability of each class given the feature values and predicts the class with the highest probability.	[37]
Transformer	A deep learning architecture that uses self-attention mechanisms to process sequential data. It replaces traditional recurrent connections with attention layers that can directly model relationships between all positions in a sequence, allowing parallel processing and better handling of long-range dependencies.	[53]

**Figure 5:**
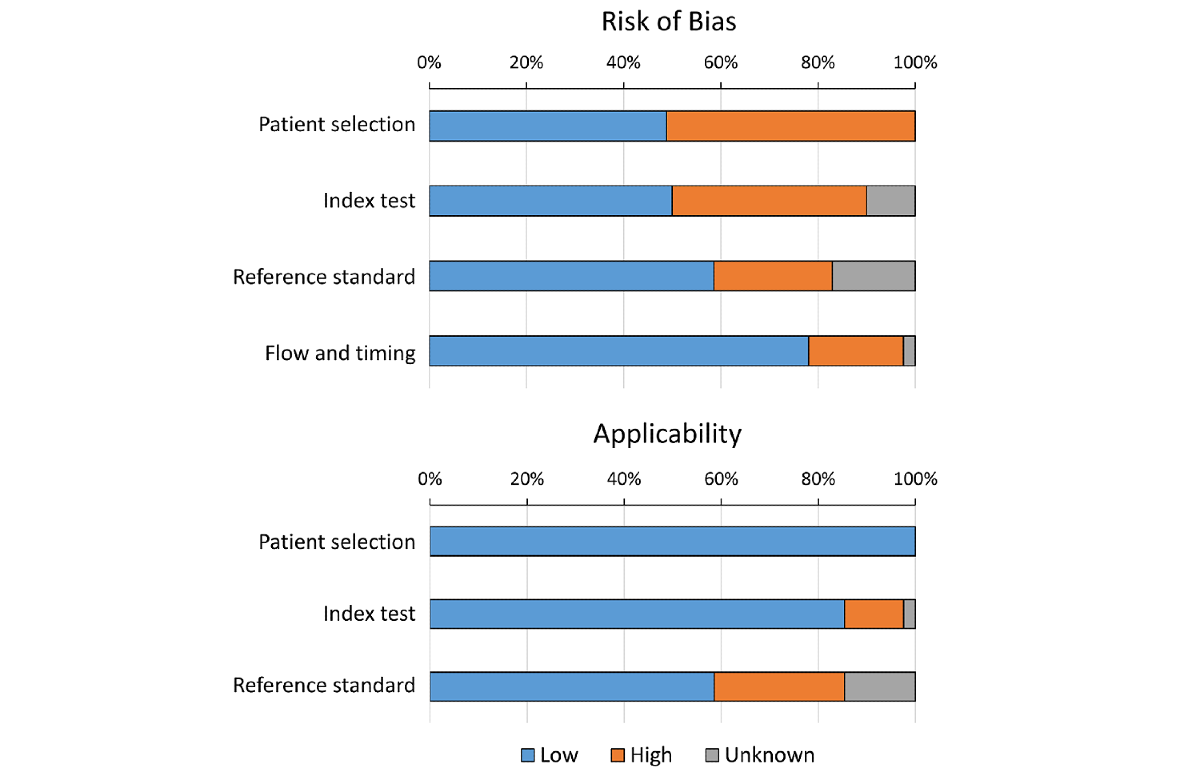
Quality assessment summary plot for risk of bias (top) and applicability concerns (bottom). Quality assessment was conducted with a modified version of the Quality Assessment of Diagnostic Accuracy Studies-2 instrument.

Quality assessment of the included studies identified several areas of potential bias. Seventeen studies showed a high risk of bias in patient selection, mainly due to insufficient description of the consecutive or random sampling process for the test set. Index test domain was marked as high risk in 11 studies, mainly due to the lack of an independent test set. Studies that used k-fold cross-validation or leave-one-out method, ensuring no overlap of samples from the same subject in both the training and validation sets, were considered low risk. One study included data beyond lung sounds in the classification model, making the index test results dependent on patient characteristics. Studies that did not specify the test set were marked as unknown. Regarding reference standards, labels assigned by at least 2 independent personnel or based on objective measures were considered reliable. A total of 8 studies were marked as high risk, while 7 studies that did not describe their labeling methods were marked as unknown. Most studies did not have issues with flow and timing (Figure 5 and Multimedia Appendix 5 [14-54]).

## Discussion

### Principal Findings

#### Overview

This systematic literature review of studies of lung sound analysis highlighted the feasibility of AI models in classifying pediatric lung sounds with moderate to high accuracy. There has been a substantial increase in the number of studies in recent years, especially in the 2020s, reflecting advancements in computational power, availability of large datasets, and improvements in ML techniques. This review included 35 studies that varied in classification tasks, sample sizes, age ranges, lung sound databases used, and model evaluation metrics.

#### Classification Tasks and Challenges

Classification tasks in respiratory sound analysis generally involve distinguishing between different lung pathologies and lung sounds. While the ultimate goal of respiratory physical examination, including auscultation, is to reach a clinical diagnosis, labeling lung sounds based on respiratory diagnoses presents significant challenges, particularly in children. Many respiratory conditions overlap; for example, bronchiolitis and pneumonia can coexist in a child, or an asthma exacerbation may be triggered by a respiratory infection such as viral pneumonia.

Since no single diagnostic test can definitively differentiate between all overlapping conditions, objective criteria are often used to standardize labeling. For instance, pneumonia diagnosis may rely on chest x-ray findings as part of an operational definition. Similarly, when assessing disease severity, physicians often use clinical scoring systems such as the community-acquired pneumonia score for pneumonia [56]. In some cases, more objective measures are available, such as using forced expiratory volume in one second % predicted to evaluate the severity of CF [29]. These standardized approaches help ensure more consistent and objective labeling in classification tasks.

#### Evaluation Methods and Performance Metrics

Although some studies had a separate external validation set or a prospective validation set, in many cases, a separate test set was not specified, limiting the reliability of their results. Furthermore, in many studies, not all the necessary evaluation metrics were specified. According to the Standards for Reporting of Diagnostic Accuracy Studies (STARD) 2015 guidelines [57], cross-tabulation of the index test results (or their distribution) by the results of the reference standard should be provided. STARD for AI-centered diagnostic tests (STARD-AI) is still under development [58], but a confusion matrix is needed to give a fair evaluation of an AI-centered diagnostic test, as a single performance metric can be influenced by not only the performance of the index test but also the distribution of the samples with and without the diagnosis. If the study failed to provide a confusion matrix, we reconstituted one from the distribution of the test population of the reference standard, and sensitivity and specificity. However, in our review, we have found that many tests did not provide enough metrics to form a confusion matrix.

#### Dataset Quality and Availability

Significant variation was observed in the quality and quantity of the datasets used across studies. Most studies used small, institution-specific datasets, limiting the generalizability of the findings. Additionally, there was a lack of standardized data collection and annotation protocols. Additionally, there was a lack of standardized data collection and annotation protocols. Particularly, the stethoscopes used to collect data varied widely, limiting their generalizability. The only publicly available pediatric database, Shanghai Pediatric Respiratory Sound Database, came from a single center [37]. A few multicenter datasets were used in different studies, but the datasets were not available for public use. To develop a more robust and generalizable pediatric lung sound analysis model, a large-scale multicenter pediatric lung sound database is crucial.

#### Feature Extraction Methods

The most frequently used feature extraction methods in the reviewed studies were MFCC, Mel-spectrogram, and Fourier transform-based methods. Mel-spectrogram and STFT are both time-frequency representations of an audio signal, providing information about the signal’s frequency content over time [59]. They involve dividing the signal into short frames and applying the Fourier transform to each frame. However, while STFT provides a linear frequency scale, Mel-spectrogram applies a Mel-scale filter bank based on human perception of pitch. This emphasizes the lower frequencies that humans are more sensitive to, making Mel-spectrogram more perceptually relevant. MFCC is derived from the Mel-spectrogram by applying additional processing steps, such as taking the logarithm and applying the discrete cosine transform, resulting in a compressed feature vector that captures the overall shape of the spectral envelope [60]. In adult-based lung sound classification studies, new features such as Chromagram, representation of the intensity of different pitch classes (chromas) over time, irrespective of the octave, have been explored [61].

#### ML Models

The reviewed studies used a diverse range of ML models for classifying lung sounds and diagnosing respiratory conditions. CNN and its variants, such as CNN combined with recurrent neural network and ResNet, emerged as the most popular choice, with a total of 17 studies adopting these architectures. The prevalence of CNN-based models can be attributed to their ability to automatically learn and extract relevant features from the input data, making them well-suited for processing complex signals such as lung sounds. ResNet, a deep CNN architecture with residual connections, allows for the training of much deeper networks without the vanishing gradient problem, enabling the network to capture complex hierarchical features and dependencies in lung sound data [62]. Apart from neural network-based models, SVM was also widely used, being used in 6 studies. SVMs are known for their ability to find optimal decision boundaries in high-dimensional feature spaces, making them effective in classifying lung sounds based on extracted features [63]. While other models such as Hidden Markov Models, k-nearest neighbors, ensemble models, and probabilistic classifiers were explored in some studies, the dominance of CNN-based architectures, particularly ResNet, and SVMs underscores their effectiveness in accurately classifying lung sounds and diagnosing respiratory conditions.

### Comparison With Prior Work

There is a limited number of reviews on lung sound analysis using ML [13,64-66]. Compared with prior work in adult populations, this study provides a unique focus on pediatric datasets, where challenges such as overlapping conditions and smaller dataset sizes are more pronounced. While a recent scoping review on pediatric asthma diagnosis using lung sound analysis was published, this study expands the scope by including various lung pathologies and sound classification tasks [67].

### Strengths

Our systematic review and meta-analysis provide a comprehensive review of ML used in pediatric lung sound analysis, offering an evidence base for future research in this field. The study synthesizes findings from multiple studies, identifying common trends, challenges, and gaps in research. Additionally, this review emphasizes the need for standardized reporting guidelines and the development of multicenter pediatric lung sound datasets.

### Limitations

The study has some limitations. First, we have only searched for articles in English. Second, due to the heterogeneity of the classification tasks of the included studies and the lack of essential results in some studies, meta-analysis was possible for only parts of the data. Third, the lack of standard guidelines in AI-centered diagnostic studies undermines the objectivity of quality assessment in our study. To address this limitation, we have made our best efforts to modify the QUADAS-2 to fit AI-centered diagnostics. These weaknesses may be overcome in the future when STARD-AI is published and upcoming studies conform to this guideline [58], and when QUADAS-AI is also published for evaluating these studies [68]. In the meanwhile, since AI in medical imaging is the leading field of AI-based diagnostics, we can draw from existing guidelines on reporting AI in medical imaging [69].

### Future Directions

While ML models have shown high accuracy in the classification of lung sounds, several challenges remain in implementing the models into clinical practice. First, the black-box nature of ML models limits clinical interpretability. Explainable AI techniques that provide visual insights into the model would be useful for clinical decision-making and sharing information with patients. Second, for the practical use of these models, stethoscopes equipped with real-time analysis models need to be developed. Technical issues must be resolved to implement lung sound classification models in clinical practice and electronic health record systems. Last, the clinical value of these studies has not been shown in any studies. Clinical trials to validate the efficacy of ML models in clinical settings, such as better diagnostic accuracy or faster decision-making in the clinic, should be implemented according to guidelines [70]. Before these models are used in clinical and household settings, ethical and privacy issues must be addressed [71,72].

In conclusion, pediatric lung sound analysis can be performed with high accuracy. However, due to the lack of standard guidelines, there is significant heterogeneity in the reported studies. Standardization in this emerging field is necessary. Future research should prioritize robust designs with external validation, detailed descriptions of model development, and comprehensive performance results. With the development of real-time analysis tools that can be deployed in various clinical settings, pediatric lung sound analysis has the potential to improve respiratory care, providing timely and accurate diagnoses, and ultimately enhancing clinical outcomes for pediatric patients.

## Data Availability

The data supporting this systematic review are extracted from publicly available studies included in the review.

## Appendix

Multimedia Appendix 1 Preferred Reporting Items for Systematic Reviews and Meta-Analysis for Diagnostic Test Accuracy Studies (PRISMA-DTA) checklist.

Multimedia Appendix 2 Database and search queries used in this study.

Multimedia Appendix 3 Characteristics of studies included in this review on pediatric lung sound analysis.

Multimedia Appendix 4 Details of databases used by studies included in this review on pediatric lung sound analysis.

Multimedia Appendix 5 Quality assessment results of studies included in this review.

## Abbreviations

AI: artificial intelligence
CF: cystic fibrosis
CNN: convolutional neural network
FN: false negative
FP: false positive
HMM: hidden Markov model
MFCC: Mel-frequency cepstral coefficients
ML: machine learning
PRISMA-DTA: Preferred Reporting Items for Systematic Reviews and Meta-Analysis for Diagnostic Test Accuracy Studies
QUADAS-2: Quality Assessment of Diagnostic Accuracy Studies (Version 2)
ResNet: residual network
STARD: Standards for Reporting of Diagnostic Accuracy Studies
STFT: short-time Fourier transform
SVM: support vector machine
TN: true negative
TP: true positive
