# Supplementary material for: Artificial Intelligence Models for Pediatric Lung Sound Analysis: Systematic Review and Meta-Analysis
Source: J Med Internet Res. 2025 Apr 18;27:e66491. doi: 10.2196/66491 (PMC12048790; doi:10.2196/66491)
Supplement: Multimedia Appendix 2 [file jmir_v27i1e66491_app2.docx]

**Multimedia Appendix 2**

Database and search queries used in this study.

| **DB** | **Search** | **Query** | **Results** |
| --- | --- | --- | --- |
| PubMed | 1 | "Child, Preschool"[Mesh] OR "Child"[Mesh] OR "Adolescent"[Mesh] OR "Pediatrics"[Mesh] OR "Infant"[Mesh] OR "Schools"[Mesh] | 4,235,732 |
|  | 2 | "child preschool"[Title/Abstract] OR "Child"[Title/Abstract] OR "pediat*"[Title/Abstract] OR "Schools"[Title/Abstract] OR "preschool*"[Title/Abstract] OR "Children"[Title/Abstract] OR "adolescen*"[Title/Abstract] OR "youth*"[Title/Abstract] OR "teen*"[Title/Abstract] OR "childhood"[Title/Abstract] OR "paediat*"[Title/Abstract] OR "Neonatology"[Title/Abstract] OR "Perinatology"[Title/Abstract] OR "newborn*"[Title/Abstract] OR "neonate*"[Title/Abstract] OR "infant*"[Title/Abstract] OR "juvenile*"[Title/Abstract] OR "secondary education*"[Title/Abstract] OR "primary school*"[Title/Abstract] OR "secondary school*"[Title/Abstract] OR "baby*"[Title/Abstract] OR "babies"[Title/Abstract] OR "kid"[Title/Abstract] OR "kids"[Title/Abstract] OR "boy"[Title/Abstract] OR "girl"[Title/Abstract] OR "toddler*"[Title/Abstract] OR "first grader*"[Title/Abstract] OR "second grader*"[Title/Abstract] OR "third grader*"[Title/Abstract] OR "fourth grader*"[Title/Abstract] OR "fifth grader*"[Title/Abstract] OR "sixth grader*"[Title/Abstract] OR "seventh grader*"[Title/Abstract] OR "highschool*"[Title/Abstract] OR "prepuberty"[Title/Abstract] OR "puberty"[Title/Abstract] | 2,992,513 |
|  | 3 | "Respiratory Sounds"[Mesh] OR "auscultation"[Mesh] OR "Lung"[Mesh] | 339,480 |
|  | 4 | "respiratory sound*"[Title/Abstract] OR "auscultation"[Title/Abstract] OR "Lung"[Title/Abstract] OR "lung sound*"[Title/Abstract] OR "lung auscultation"[Title/Abstract] OR "respiratory tract sound*"[Title/Abstract] OR "abnormal respiratory sound"[Title/Abstract] OR "adventitious sound*"[Title/Abstract] OR "wheez*"[Title/Abstract] OR "crackle*"[Title/Abstract] OR "rale*"[Title/Abstract] OR "stridor*"[Title/Abstract] OR "rhonch*"[Title/Abstract] OR "breath sound"[Title/Abstract] OR "breathing sound*"[Title/Abstract] OR "pleural rub*"[Title/Abstract] OR "rale*"[Title/Abstract] OR "pulmonary auscultation"[Title/Abstract] OR "respiration sound*"[Title/Abstract] OR "chest auscultation"[Title/Abstract] OR "thoracic sounds"[Title/Abstract] OR (("acoust"[All Fields] OR "acoustical"[All Fields] OR "acoustically"[All Fields] OR "acoustics"[MeSH Terms] OR "acoustics"[All Fields] OR "acoustic"[All Fields]) AND "respiratory analysis"[Title/Abstract]) OR "auscultatory findings"[Title/Abstract] | 824,697 |
|  | 5 | "machine learning"[Mesh] OR "artificial intelligence"[Mesh] OR "Supervised Machine Learning"[Mesh] OR "Unsupervised Machine Learning"[Mesh] OR "Deep Learning"[Mesh] | 216,992 |
|  | 6 | "machine learning"[Title/Abstract] OR "artificial intelligence"[Title/Abstract] OR "Supervised Machine Learning"[Title/Abstract] OR "Unsupervised Machine Learning"[Title/Abstract] OR "Deep Learning"[Title/Abstract] OR "neural network*"[Title/Abstract] OR "artificial intelligence"[Title/Abstract] OR "transfer learning"[Title/Abstract] OR "supervised learning"[Title/Abstract] OR "unsupervised learning"[Title/Abstract] OR "Hierarchical Learning"[Title/Abstract] OR "AI"[Title/Abstract] OR "Computer Reasoning"[Title/Abstract] OR "Machine Intelligence"[Title/Abstract] OR "Computational Intelligence"[Title/Abstract] OR "computer vision system*"[Title/Abstract] OR "Knowledge Acquisition"[Title/Abstract] OR "knowledge representation*"[Title/Abstract] OR "self-supervised learning"[Title/Abstract] OR "transformer"[Title/Abstract] OR "support vector machine"[Title/Abstract] OR "nearest neighbo*"[Title/Abstract] OR "naive bayes"[Title/Abstract] OR "Markov model"[Title/Abstract] OR "decision tree"[Title/Abstract] OR "random forest"[Title/Abstract] OR "large language model"[Title/Abstract] | 410,056 |
|  | 7 | (#1 OR #2) AND (#3 OR #4) AND (#5 OR #6) | **683** |
| Ovid Medline | 1 | exp Child, Preschool/ or exp Child/ or exp Adolescent/ or exp Pediatrics/ or exp Infant/ or exp Schools/ | 4,237,877 |
|  | 2 | (child or pediatrics or Schools or preschool* or Children or adolescen* or youth* or teen* or childhood or paediat* or Neonatology or Perinatology or newborn* or neonate* or infant* or juvenile* or secondary education* or Primary School* or secondary School* or baby* or babies or kid or kids or boy or girl or toddler* or first-grader* or second-grader* or third-grader* or fourth-grader* or fifth-grader* or sixth-grader* or seventh-grader* or highschool* or prepuberty or puberty).ab,ti. | 2,761,591 |
|  | 3 | #1 or #2 | 5,030,141 |
|  | 4 | exp Respiratory Sounds/ or exp Auscultation/ or exp Lung/ | 339,590 |
|  | 5 | (respiratory sounds or auscultation or lung sound* or lung auscultation or respiratory tract sound or abnormal respiratory sound or adventitious sound* or wheez* or crackle* or rale* or stridor* or rhonch* or breath sound or Breathing Sound* or Pleural Rub* or Rale* or pulmonary auscultation or respiration sound or chest auscultation or thoracic sounds or acoustic respiratory analysis or auscultatory findings).ab,ti. | 30,112 |
|  | 6 | #4 or #5 | 358,296 |
|  | 7 | exp Machine Learning/ or exp Artificial Intelligence/ or exp Supervised Machine Learning/ or exp Unsupervised Machine Learning/ or exp Deep Learning/ | 217,653 |
|  | 8 | (machine learning or artificial intelligence or Supervised Machine Learning or Unsupervised Machine Learning or Deep Learning or neural network* or artificial intelligence or transfer learning or supervised learning or unsupervised learning or Hierarchical Learning or 'AI' or Computer Reasoning or Machine Intelligence or Computational Intelligence or Computer Vision System* or Knowledge Acquisition or Knowledge Representation* or self-supervised learning or transformer or support vector machine or nearest neighbo* or naive bayes or Markov model or decision tree or random forest or large language model).ab,ti. | 386,299 |
|  | 9 | #7 or #8 | 465,669 |
|  | 10 | #3 and #6 and #9 | **321** |
| EMBASE | 1 | preschool child'/exp OR 'child'/exp OR 'adolescent'/exp OR 'pediatrics'/exp OR 'infant'/exp OR 'school'/exp | 4,923,102 |
|  | 2 | child:ab,ti OR pediatrics:ab,ti OR school:ab,ti OR preschool*:ab,ti OR children:ab,ti OR adolescen*:ab,ti OR youth*:ab,ti OR teen*:ab,ti OR childhood:ab,ti OR paediat*:ab,ti OR neonatology:ab,ti OR perinatology:ab,ti OR newborn*:ab,ti OR neonate*:ab,ti OR infant*:ab,ti OR juvenile*:ab,ti OR 'secondary education*':ab,ti OR 'primary school*':ab,ti OR 'secondary school*':ab,ti OR baby*:ab,ti OR babies:ab,ti OR kid:ab,ti OR kids:ab,ti OR boy:ab,ti OR girl:ab,ti OR toddler*:ab,ti OR 'first grader*':ab,ti OR 'second grader*':ab,ti OR 'third grader*':ab,ti OR 'fourth grader*':ab,ti OR 'fifth grader*':ab,ti OR 'sixth grader*':ab,ti OR 'seventh grader*':ab,ti OR highschool*:ab,ti OR prepuberty:ab,ti OR puberty:ab,ti | 3,750,102 |
|  | 3 | 'abnormal respiratory sound'/exp OR 'auscultation'/exp OR 'lung'/exp | 549,197 |
|  | 4 | lung sound*':ab,ti OR 'lung auscultation':ab,ti OR 'respiratory tract sound':ab,ti OR 'abnormal respiratory sound':ab,ti OR 'adventitious sound*':ab,ti OR wheez*:ab,ti OR crackle*:ab,ti OR stridor*:ab,ti OR rhonch*:ab,ti OR 'breath sound':ab,ti OR 'breathing sound*':ab,ti OR 'pleural rub*':ab,ti OR rale*:ab,ti OR 'pulmonary auscultation':ab,ti OR 'respiration sound':ab,ti OR 'chest auscultation':ab,ti OR 'thoracic sounds':ab,ti OR 'acoustic respiratory analysis':ab,ti OR 'auscultatory findings':ab,ti | 47,056 |
|  | 5 | 'machine learning'/exp OR 'artificial intelligence'/exp OR 'supervised machine learning'/exp OR 'unsupervised machine learning'/exp OR 'deep learning'/exp | 582,564 |
|  | 6 | neural network*':ab,ti OR 'artificial intelligence':ab,ti OR 'transfer learning':ab,ti OR 'supervised learning':ab,ti OR 'unsupervised learning':ab,ti OR 'hierarchical learning':ab,ti OR 'ai':ab,ti OR 'computer reasoning':ab,ti OR 'machine intelligence':ab,ti OR 'computational intelligence':ab,ti OR 'computer vision system*':ab,ti OR 'knowledge acquisition':ab,ti OR 'knowledge representation*':ab,ti OR 'self-supervised learning':ab,ti OR transformer:ab,ti OR 'support vector machine':ab,ti OR 'nearest neighbo*':ab,ti OR 'naive bayes':ab,ti OR 'markov model':ab,ti OR 'decision tree':ab,ti OR 'random forest':ab,ti OR 'large language model':ab,ti | 363,089 |
|  | 7 | #1 OR #2 | 13,211,319 |
|  | 8 | #3 OR #4 | 560,436 |
|  | 9 | #5 OR #6 | 736,798 |
|  | 10 | #7 AND #8 AND #9 | **631** |
| Web of Scinece | 1 | TS=(child or pediatrics or Schools or preschool* or Children or adolescen* or youth* or teen* or childhood or paediat* or Neonatology or Perinatology or newborn* or neonate* or infant* or juvenile* or secondary education* or Primary School* or secondary School* or baby* or babies or kid or kids or boy or girl or toddler* or first-grader* or second-grader* or third-grader* or fourth-grader* or fifth-grader* or sixth-grader* or seventh-grader* or highschool* or prepuberty or puberty) | 4281844 |
|  | 2 | TS=(respiratory sounds or auscultation or lung sound* or lung auscultation or respiratory tract sound or abnormal respiratory sound or adventitious sound* or wheez* or crackle* or rale* or stridor* or rhonch* or breath sound or Breathing Sound* or Pleural Rub* or Rale* or pulmonary auscultation or respiration sound or chest auscultation or thoracic sounds or acoustic respiratory analysis or auscultatory findings) | 40,196 |
|  | 3 | TS=(machine learning or artificial intelligence or Supervised Machine Learning or Unsupervised Machine Learning or Deep Learning or neural network* or transfer learning or supervised learning or unsupervised learning or Hierarchical Learning or AI or Computer Reasoning or Machine Intelligence or Computational Intelligence or Computer Vision System* or Knowledge Acquisition or Knowledge Representation* or self-supervised learning or transformer or support vector machine or nearest neighbo* or naïve bayes or Markov model or decision tree or random forest or large language model) | 2,281,292 |
|  | 4 | #3 AND #2 AND #1 | **298** |
| IEEExplore | 1 | (child OR pediatric OR school OR preschool OR children OR adolescen* OR youth OR teen OR childhood OR neonatal OR perinatal OR newborn OR neonate OR infant OR juvenile OR "secondary education" OR "primary school" OR "secondary school" OR baby OR babies OR kid OR kids OR boy OR girl OR toddler OR "first-grader" OR "second-grader" OR "third-grader" OR "fourth-grader" OR "fifth-grader" OR "sixth-grader" OR "seventh-grader" OR highschool OR prepuberty OR puberty) | 1139283 |
|  | 2 | (respiratory sounds OR auscultation OR "lung sound*" OR "lung auscultation" OR "respiratory tract sound" OR "abnormal respiratory sound" OR "abnormal respiratory sounds" OR "adventitious sound*" OR wheez* OR crackle OR crackles OR rale OR rales OR stridor OR rhonch* OR "breath sound" OR "breathing sound" OR "breathing sounds" OR "pleural rub" OR "pulmonary auscultation" OR "respiration sound" OR "chest auscultation" OR "thoracic sounds" OR "acoustic respiratory analysis" OR "auscultatory findings") | 3,838 |
|  | 3 | (machine learning OR "artificial intelligence" OR "supervised machine learning" OR "unsupervised machine learning" OR "deep learning" OR "neural network*" OR "transfer learning" OR "supervised learning" OR "unsupervised learning" OR "hierarchical learning" OR AI OR "computer reasoning" OR "machine intelligence" OR "computational intelligence" OR "computer vision system*" OR "knowledge acquisition" OR "knowledge representation*" OR "self-supervised learning" OR transformer OR "support vector machine" OR "nearest neighbo*" OR "naïve bayes" OR "Markov model" OR "decision tree" OR "random forest" OR "large language model") | 1,197,420 |
|  | 4 | #3 AND #2 AND #1 | **556** |
