# Supplementary material for: Artificial Intelligence Models for Pediatric Lung Sound Analysis: Systematic Review and Meta-Analysis
Source: J Med Internet Res. 2025 Apr 18;27:e66491. doi: 10.2196/66491 (PMC12048790; doi:10.2196/66491)
Supplement: Multimedia Appendix 3 [file jmir_v27i1e66491_app3.docx]

**Multimedia Appendix 3**

Characteristics of studies included in this review on pediatric lung sound analysis.

| Author, Year | Database | Sample size | Task | Train/Test size | Feature extraction | Model | Performance |
| --- | --- | --- | --- | --- | --- | --- | --- |
| Forkheim, 1995 (14) | Private: from Respiration Acoustics Laboratory at Children's Hospital, Winnipeg | 710 samples | Wheeze detection | Training set: 242,  Test set 1: 233 from same patient group as training set;  Test set 2: 235 from different patient group | Raw signal data + Fourier transform data scaled to 247 values | Backpropagation neural network, Radial basis function (RBF) network, Self-organizing map (SOM), Learning vector quantization (LVQ) | RBF: 94.5% accuracy overall on test sets, LVQ: 94.5% accuracy overall on test sets |
| Rietveld, 1999 (15) | Private: 50 school-age asthma children, 10 controls | 48 samples of 2 second duration each | Three subject groups classification: asthma exacerbation, asthma remission, healthy control | Train:72, Test: 12 samples | Fourier power spectra computed from 3-second intervals representing a full breath cycle,  Frequency range 100-1300 Hz divided into 26 bands of ~46 Hz each | Self-classifying competitive neural network, Feedforward neural network with supervised learning | 95% training vectors classified correctly; 43% test vectors classified correctly |
| Emmanouilidou, 2012 (16) | Private: outpatient pediatric clinic in Kathmandu, Nepal | 28 recordings of 15-sec duration (10 normal, 10 wheeze, 8 crackle cases) | 2-class: normal / abnormal classification  3-class: normal / crackle / wheeze sounds classification | 10-fold cross validation (Train 90%, test 10%) | Cortical Model:  Auditory spectrogram using 128 cochlear filters  Rate-Scale-Frequency features from spectrogram (2-class: rate filters covering 0.5-32Hz, 3-class: 10 rate filters (40-256Hz) and 7 scale filters (0.125-8 c/o) | SVM with RBF kernels | 2-class: sensitivity 89.44%, specificity 80.50%, AUC 0.9217  3-class: Volume Under the ROC Surface (VUS) 0.601 |
| Khan, 2012 (17) | Private: lung sound recording from 40 subjects (20 normal, 20 with bronchitis) | Not mentioned | Normal / bronchitis detection | Not mentioned | Mel-frequency cepstral coefficients (MFCC) with 18 coefficients | Feed forward neural network with 1 hidden layer (18 neurons) | Normal: 90%, Bronchitis: 90%  - accuracy 90% |
| Jin, 2014 (18) | Private: 5 healthy and 19 pathological subjects | 89 normal segments (59 inspiratory, 30 expiratory), 150 CAS segments (67 inspiratory, 83 expiratory) | Normal and continuous adventitious sounds (CAS) classification | Leave-one-out (LOO) method | Based on instantaneous kurtosis of narrowband signals, discriminating function using ratio of short-term and long-term autoregressive averaging, and sample entropy and histogram distortion | Support Vector Machine (SVM) classifier with RBF and linear kernels | Inspiratory sound 98.3% accuracy, Expiratory sound 97.6% accuracy |
| Mazic, 2015 (19) | Private: 16 children at the General Hospital of Dubrovnik | 45 phono-pneumograms (PPG), each 10 seconds long (269 wheeze segments from 21 PPG, 2388 non-wheeze segments from 12 PPG) | Wheeze detection | 21 PPG for training/validation,  24 PPG for testing | MFCC, kurtosis, Renyi entropy | Two-level SVM cascade classifier | 100% reliability (true positive rate * true negative rate) |
| Milicevic, 2016 (20) | Internet (INT) databases, Private: General Hospital of Dubrovnik (DGH) | INT data: 1026 wheeze and 1374 non-wheeze segments, DGH data: 369 wheeze and 495 non-wheeze segments (100ms each) | Wheeze / normal breathing classification | 10-fold cross validation | MFCC: 15 coefficients (100-1500Hz) for INT, 12 coefficients (100-1000Hz) for DGH, Statistical features (Renyi entropy, Kurtosis, SF, Skewness, MCI, SD, f50/f90), Filter Bank Energy Coefficients | SVM, k-Nearest Neighbor (k-NN), Neural Network, Random Forest, Logistic Regression, Naive Bayes | Best accuracy with MFCCs 99% (SVM, k-NN) for both INT and DGH data, Best accuracy with statistical features 93.62%(INT), 91.77%(DGH) using neural network |
| Khan, 2017 (21) | Private: children hospitals of Pusad, Digras and Nagpur in the state of Maharashtra India | 254 sounds (split equally into 127 normal and 127 pathological recordings) | Normal / adventitious lung sounds classification | 40% for training, 60% for classification | Short time Fourier transform (STFT), singular value decomposition for feature reduction | k-NN, SVM with linear kernel | k-NN: sensitivity 90.9%, specificity 92.20%, accuracy 91.55%  SVM: sensitivity, specificity, and accuracy of 92.20% |
| Emmanouilidou. 2018 (22) | Pneumonia Etiology Research for Child Health (PERCH) study | Final dataset over 250 hours of lung sounds | Abnormal / normal lung sound classification | 10-fold cross validation (Subjects in training and testing were mutually exclusive.) | Cochlear filter bank analysis followed by spectral sharpening and modulation filtering, Tensor Singular Value Decomposition used for dimensionality reduction | SVM classifier with RBF kernel | Accuracy 86.7% |
| Mohamed, 2018 (23) | Private: 60 cases with chest infection at the paediatric emergency department of Geneva University Hospital | 424 recordings segments (48 patients and 5 controls) | Bacterial pneumonia classification | Not mentioned | Mel spectrogram | Combining Convolutional Neural Network (CNN) and Hidden Markov Model (HMM) | Area Under the Curve (AUC) 0.89 |
| Gouda, 2019 (24) | Private: 446 respiratory sounds collected from Alexandria University Children Hospital | Total 446 samples. 300 sounds (100 each of normal, wheeze, stridor) for training and testing, Additional 146 wheeze sounds for validation | Normal/ wheeze/ stridor classification | 300 sounds: 70% for training, 30% for testing, additional 146 sounds: validation set | Discrete Wavelet Transform (DWT), STFT, MFCC | Artificial Neural Network (ANN), SVM - polynomial kernel, KNN, Naive Bayes (NB) | Overall accuracy 100% (DWT + ANN),  Wheeze detection accuracy 100% (MFCC + ANN),  Validation on 146 wheeze sounds accuracy 100% (MFCC + ANN) |
| Grzywalski, 2019 (25) | Private: 50 pediatric patients at the Department of Paediatric Pulmonology, Karol Jonscher University Hospital in Poznan | 522 recordings | Normal/ wheeze/ ronchi/ coarse crackles/ fine crackles classification | Not mentioned | Spectrogram | Neural network architecture composed of convolutional layers to detect local correlations in signal  Recurrent layers to capture long-term dependencies like breathing cycles | F1 score  Fine crackles 83.9%  coarse crackles 47.1%  rhonchi 72.0%  wheezes 66.4% |
| Liu L, 2019 (26) | Private: 12 children from a Chinese women and children hospital and School of Nursing at Northern Illinois University | 120 wav files - 30 each of asthma, croup, pneumonia, and normal breath sounds | Asthma/ croup/ pneumonia/ normal classification | Not mentioned | Linear Predictive Coding (LPC), Linear Predictive Cepstral Coefficients (LPCC), MFCC | KNN, ANN | MFCC with ANN: 83.3% accuracy |
| Liu R, 2019 (27) | Private: 508 recordings from 222 pediatric subjects  Public: ICBHI 2017 | 1094 segments (561 with adventitious sounds, 533 without) | With/ without adventitious sounds classification | Training set 75%, test set 25% | Log Mel-filterbank (LMFB), 3D representation | CNN | Pediatric test set: 69.72% accuracy |
| Kotb, 2020 (28) | Private: chest sound records from 116 children | 464 chest sound records (261 for training, 203 for validation) | Normal vesicular/ wheezes/ crackles/ bronchial breathing classification | 261 recordings for training models, 203 recordings for validating models | MFCC, PPG | HMM | HMM with MFCCs and PPG wave shape: 98.7% accuracy |
| Karimizadeh, 2021 (29) | Private: 37 cystic fibrosis (CF) patients | 209 multichannel lung sound samples (77 normal, 33 mild, 39 moderate, 60 severe) | Severity of CF (normal/ mild/ moderate/ severe) classification | Leave-one-sample-out, 5-fold cross-validation, leave-one-subject-out methods | Expiration to inspiration lung sound power ratio in different frequency bands for large airways, upper airways and peripheral airways | SVM, ANN, Decision tree, NB | For all severity levels: Neural Network with 89.05% average accuracy using all features |
| Kuo, 2021 (30) | Private: 95 children (63 healthy, 32 with asthma) | Not mentioned | Healthy / wheezing classification | Not mentioned | Respiratory rate, sound index, breathing cycle period, inspiratory/expiratory durations, maximum peak frequency, wheezing duration and frequency from lung sound spectrogram | Radial basis function neural network (RBFNN) | F1-score 95.2%, Accuracy 96.8%, Precision 96.8%, Sensitivity 93.8% |
| Liu, 2021 (31) | Private: 12 children from a Chinese women and children hospital and School of Nursing at Northern Illinois University | 120 wave files - 30 each of asthma, croup, pneumonia and normal breath sounds | Asthma/ croup/ pneumonia/ normal classification | Not mentioned | LPC, LPCC, MFCC, Time-varying LPC (TVLPC) | KNN, ANN, HMM | KNN: 80% accuracy with MFCC features, HMM: Up to 80% accuracy with TVLPC features, 10 training loops, 70% training data |
| Cheng, 2022 (32) | Private: 93 locally collected subjects  + R.A.L.E Lung sounds database | 73 single-label samples (45 normal, 10 crackles, 18 wheezes) | Normal/ crackles/ wheezes classification | 5-fold cross-validation | Patent-pending numeric features involving Mean Crossing, Tonality | SVM | Sensitivity 91%, Specificity 95% |
| Gelman, 2022 (33) | Private: Regional Children’s Clinical Hospital of Perm and the polyclinic of Perm State Medical University | 951 patients (232 in exacerbation, 309 in remission, and 410 in incomplete remission), 167 healthy volunteers | Asthma/ healthy classification | Training: 374 asthmatic, 146 healthy, Test: 577 asthmatic, 21 healthy. | Spectral bandwidth,  spectral centroid, zero-crossing rate, spectral roll-off,  and chroma feature | Sequential neural network | Accuracy 90% for patients, 87% for healthy, ±1% when test set altered |
| Kim, 2022 (34) | Private: University hospitals in Korea | 76 patients | Wheeze detection | Train 80%, test 20% | Mel spectrogram, gender and age data passed through the MLP model consisting of 8 and 16 nodes | 34-layer residual network with the convolutional block attention module for audio data and multilayer perceptron layers for tabular data | Accuracy 91.2%, AUC 89.1%, precision 94.4%, recall 81%, and F1-score 87.2% |
| Ma, 2022 (35) | Public: SPRSound | 292 patients | Task 1-1: Normal/ adventitious, Task 1-2: normal/ ronchi/ wheeze/ stridor/ coarse crackle/ fine crackle/ wheeze and crackle,  Task 2-1: normal/ adventitious/ poor quality,  Task 2-2: normal/CAS/ DAS/ CAD&DAS/ poor quality | Train: 6,656 events, 251 participants.  Testing-1:  1,004 respiratory sound events.  Testing-2:  1,429 respiratory sound events from 41 participants. | Spectrogram, CenterCrop, Normalization | DenseNet169 | Total score*  Task 1-1 89.0%, Task 1-2 90.9%,  Task 2-1 83.8%, Task 2-2 67.3% |
| Nguyen, 2022 (36) | Private: Children’s National Hospital ED | 1095 recordings (644 clear, 451 wheeze) | Wheeze/ clear classification | Train/test 80%/20% based on patient | Spectrogram | ResNet-18, Harmonic Networks | Harmonic Networks: accuracy 84%, sensitivity 89%, specificity 78%. |
| Zhang Q, 2022 (37) | Public: SPRSound | 292 patients | Task 1-1, Task 1-2, Task 2-1, Task 2-2 | Train: 6,656 events, 251 participants.  Testing-1: 1,004 respiratory sound events.  Testing-2: 1,429 respiratory sound events from 41 participants | MFCC, mel spectrogram, STFT spectrogram, log-mel spectrogram | SVM, KNN, Logistic regression, NB, Decision Tree, Random Forest | Total score*  (MFCC+NB) Task 1-1 75.22%, Task 1-2 61.57%, (MFCC+SVM) Task 2-1 56.71%, Task 2-2 37.84% |
| Li, 2022 (38) | Public:  SPRSound | 292 patients | Task 1-1, Task 1-2, Task 2-1, Task 2-2 | Same as Zhang Q, 2022 | STFT, spectrogram clipping | ResNet-18, TC-ResNet | Total score*  Task 1-1 93.3%, Task 1-2 87.9%, Task 2-1 83.3%, Task 2-2 67.3% |
| Zhang L,2022 (39) | Public:  SPRSound | 292 patients | Task 1-1, Task 1-2, Task 2-1, Task 2-2 | Same as Zhang Q, 2022 | N frames for each event, feature vectors of length 136 | Feature polymerized VO | Total score*  Task 1-1 82.0%, Task 1-2 74.3%, Task 2-1 71.1%, Task 2-2 54.1% |
| Babu, 2022 (40) | Public:  SPRSound | 292 patients | Task 1-1, Task 1-2, Task 2-1, Task 2-2 | Dataset 1: 5607 events  Dataset 2: 6656  Train/Test 80%/20% | MFCC | 2DCNN | Total score*  Task 1-1 94.3%, Task 1-2 94.8%, Task 2-1 93.7%, Task 2-2 54.1% |
| Heitman, 2023 (41) | Private: Pediatric patients with and without acute respiratory infection from 5 European countries | 572 patients | “one-versus-rest” control, pneumonia, wheezing disorder, bronchiolitis | Train/test: 2 countries, 5-fold CV  External validation: 4 countries | Log-mel spectrogram | CNN audio classifier, logistic regression for each category | AUROC for control, pneumonia, wheezing disorder, bronchiolitis: 0.93, 0.75, 0.91, 0.94 |
| Hu, 2023 (42) | Public:  SPRSound + Grand Challenge 23 dataset | 292 + 95 patient | Task 1-1, Task 1-2, Task 2-1, Task 2-2 | Train/test: SPRSound, Grand Challenge 23 dataset: 2,683 records and 9,089 respiratory sound events | MFCC | Supervised Contrastive Pretraining,  MixUp Finetuning,  ResNet base | Total score*  Task 1-1 76.9%, Task 1-2 63.2%, Task 2-1 66.2%, Task 2-2 51.2% |
| Huang, 2023-1 (43) | Private:  Children CAP Lung sound dataset | 1198 lung records ((198 child× 2 position× 3 stage) | Community acquired pneumonia (CAP) diagnosis (CAP/ healthy), CAP prognosis (CAP confirmed/ improved/ recovery) classification | 10-fold CV  (Subject independent) | Log-mel spectrogram | Bilateral pulmonary audio-auxiliary model (BPAM) | CAP diagnosis: sensitivity 70.30%, specificity 50.0%  CAP prognosis: sensitivity 43.26%, specificity 39.61% |
| Huang, 2023-2 (44) | Private:  Children CAP Lung sound dataset | 1198 lung records ((198 child× 2 position× 3 stage) | CAP diagnosis (CAP/ healthy), CAP prognosis (CAP confirmed/ improved/ recovery) classification | 10-fold CV  (Subject independent) | Log-mel spectrogram | Contrastive embedding-based domain adaptation network (CEDANN) | CAP diagnosis: sensitivity 64.17%, specificity 68.05%  CAP prognosis: sensitivity 59.06%, specificity 59.55% |
| Ngo, 2023-1 (45) | Public:  SPRSound + Grand Challenge 23 dataset | 292 + 95 patient | Task 1-1, Task 1-2, Task 2-1, Task 2-2 | Same as Hu, 2023 | Continuous wavelet transformation spectrogram | Inception (CNN based) + ResNet | Total score*  Task 1-1 81.0%, Task 1-2 66.7%, Task 2-1 74.4%, Task 2-2 60.8% |
| Ngo, 2023-2 (46) | Public:  SPRSound + Grand Challenge 23 dataset | 292 + 95 patient | Task 1-1, Task 1-2, Task 2-1, Task 2-2 | Same as Hu, 2023 | Continuous wavelet transformation spectrogram | Inception (CNN based) + ResNet + others (i.e. Use Attention block, linear combination method is used in Combiner block, KL-loss and contrastive loss). | Total score*  Task 1-1 84.9%, Task 1-2 77.4%, Task 2-1 74.5%, Task 2-2 53.9% |
| Ntalampiras, 2023 (47) | Public:  SPRSound | 292 patients | Similar/ dissimilar classification | Train 80%, test 20% | Discrete wavelet transformation | Siamese Neural Network (CNN based) | Sensitivity 0.9252, specificity 0.9045, average score 0.9148, harmonic average 0.9147 |
| Park, 2023 (48) | Private: Pediatric pulmonology outpatient clinic at Seoul National University Hospital | Retrospective 1022 clips, prospective 90 clips | Task 1: normal/ abnormal  Task 2: crackles/ wheezing  Task 3: normal/ crackles  Task 4: normal/ wheezing | 10-fold CV, prospective validation 90 clips | MFCC | SVM-based ensemble model | F1 score  Retrospective (CV)  Task 1 0.8332, Task 2 0.8363, Task 3 0.7966, Task 4 0.8936  Prospective:  Task 1 0.8000, Task 2 0.6761, Task 3 0.6746, Task 4 0.8127 |
| Pessoa, 2023 (49) | Public:  SPRSound + Grand Challenge 23 dataset | 292 + 95 patient | Task 1-1, Task 1-2, Task 2-1, Task 2-2 | Same as Hu, 2023 | STFT spectrogram | 2D CNN of spectrogram + 1D CNN of raw audio | Total score*  Task 1-1 75.6%, Task 1-2 46.7%, Task 2-1 95.8%, Task 2-2 45.8% |
| TaghiBeyglou, 2023 (50) | Public:  SPRSound + Grand Challenge 23 dataset | 292 + 95 patient | Normal/ rhonchus/ wheeze/ stridor/ coarse crackle/ fine crackle/ wheeze and crackle classification | Leave-multiple-subjects-out (LMSO) validation | Mel-spectrogram | TRespNet (ResNet-50, 1D CNN to capture temporal dynamics) | Sensitivity 0.84, Specificity 0.98, average score 0.91, harmonic score 0.90, total score* 0.90 |
| Chowdhury, 2024 (51) | Private:  19 pediatric subjects (9 healthy, 10 with pneumonia) | 35 healthy, 37 pneumonia-infected ascultation samples | Healthy/ pneumonia classification | Training 80%, testing 20% | MFCC, Zero Crossing Rate, Chromagram, Root Mean Square Energy, and Mel-spectrogram | 1D CNN | Accuracy 86.36%, precision 85.71%, recall 86.96%, F1-score 86.31%, AUC 0.93 |
| Crisdayanti, 2024 (52) | Private: 675 pediatric patients from Woorisoa Children’s Hospital in South Korea  Public: ICBHI 2017 challenge dataset | 11,154 recordings from pediatric dataset (5,605 normal, 5,549 abnormal)  6,896 recordings from ICBHI dataset (3,642 normal, 3,254 abnormal) | Normal/ abnormal respiratory sound classification | 5-fold CV for the pediatric dataset,  Train 60%, test 40% for the ICBHI dataset | STFT, Mel-spectrogram, MFCC | CNN (with 3-step knowledge propagation and a self-attention mechanism) | Best accuracy 0.8755 in pediatric dataset, 0.6597 in ICBHI dataset |
| Wang, 2024 (53) | Public:  ICBHI 2017 and SPRSound datasets | 6,898 respiratory cycles from ICBHI  9,089 respiratory cycles from SPRSound | Respiratory cycle classification  ICBHI: four-category classification (normal/ crackle/ wheeze/ wheeze and crackle)  SPRSound: seven-category classification (normal/ coarse crackle/ fine crackle/ wheeze/ wheeze and crackle/ rhonchi/ stridor) | ICBHI: 6,001 cycles for training, 2,756 cycles for testing  SPRSound: 7,796 cycles for training, 2,433 cycles for testing | Overlap Fusion-based Generalized S-Transform with sliding window-based augmentation | Swin transformer | Accuracy:  0.5605 in the ICBHI 2017 dataset  0.8018 in the SPRSound dataset |
| Yeh, 2024 (54) | Public:  SPRSound | 3324 audio files | Normal/ rhonchi/ wheeze/ stridor/ coarse crackle/ fine crackle/ wheeze and crackle classification | not mentioned | Fast Fourier Transform (FFT), boundary cropping, imbalanced data augmentation | DenseNet | F-score** 0.3296, Error rate 1.36 |

* Total score = ((SE+SP)/2) + 2*SE*SP/(SE+SP))/2, where SE = sensitivity, SP = specificity.

** F-score = 2*TP/(2*TP+FP+FN), where TP = true positive, FP = false positive, FN = false negative.
