# Supplementary material for: Artificial Intelligence Models for Pediatric Lung Sound Analysis: Systematic Review and Meta-Analysis
Source: J Med Internet Res. 2025 Apr 18;27:e66491. doi: 10.2196/66491 (PMC12048790; doi:10.2196/66491)
Supplement: Multimedia Appendix 4 [file jmir_v27i1e66491_app4.docx]

**Multimedia Appendix 4**

Details of databases used by studies included in this review on pediatric lung sound analysis.

| DB | Country | Enrollment | Age | Subject / sample number | Labeling method/person | Labels | Stethoscope/Recorder | Sampling detail | Avail-ability | Studies |
| --- | --- | --- | --- | --- | --- | --- | --- | --- | --- | --- |
| SPRSound | China | Pediatric respiratory department of Shanghai Children’s Medical Center (SCMC), Shanghai Jiao Tong University affiliated hospital | Mean 5.4 years (0.2-16.2 years) | 292 participants | Custom-made sound label annotation software (SoundAnn) each record annotated by 3 physicians independently | Event level: Normal/ ronchi/ wheeze/ stridor/ coarse crackle/ fine crackle/ wheeze & crackle Record level: Normal, CAS, DAS, CAS&DAS, Poor quality | Digital stethoscope (Yunting model II Stethoscope, Yunting II) | NA | Public | (35, 37, 39, 40, 32, 45, 46, 49, 50, 53, 54) |
| ICBHI | Portugal, Greece | School of Health Sciences, University of Aveiro (ESSUA) Aristotole University of Thessaloniki (AUTH) | 4.8 ± 4.6 years (child participants) | 49 children (total 126 participants) | ESSUA: 1 medical expert (2 respiratory physiotherapists and 1 medical doctor) AUTH: 3 experienced physicians | Normal/ wheezes/ crackles/ both | Welch Allyn 5079-400, 3M Littman 3200, microphone inserted in 3M Littmann Classic II SE, or air-electret microphones | resampled to 4kHz | Public | (27, 48, 52, 53) |
| Liu L, 2019 | China, USA | A Chinese women and children hospital and School of Nursing at Northern Illinois University | 2 months - 6 years | 12 audio recordings - 120 wav files | NA | Asthma/ croup/ pneumonia/ normal | Multi-channel digital recorder | 44.1kHz with the resolution of 16bits | Private | (25, 31) |
| CCAP-LSD | China | Children with community acquired pneumonia in four hospitals in China | mean 3.32 years | 198 children | Medical imaging as diagnostic gold standard | CAP confirmed/ improvement/ recovery | 3M wireless stethoscope | NA | Private | (43, 44) |
| Forkheim, 1995 | Canada | Respiration Acoustics Laboratory at the Children's Hospital Winnipeg | NA | 710 patterns | "Trained ear" | Wheeze/ non-wheeze | NA | 5KHz, 0.1 second intervals | Private | (14) |
| Rietveld, 1999 | Netherlands | Asthmatic enrolled via family doctors, normal control enrolled via advertisements | 7-18 years (mean 12.1, SD 2.9) | 60 children (50 with asthma and 10 healthy controls) | Severity of asthma evaluated in terms of consensus of British lung physicians | Asthma exacerbation/ asthma remission / healthy control | Electret microphone placed over suprasternal notch | 24kHz | Private | (15) |
| Emmanouilidou, 2012 | Nepal | Outpatient pediatric clinic in Kathmandu, Nepal | Young children | 28 recordings (10 normal/ 10 wheeze/ 8 crackle) | Two physicians annotated all cases | 2-class: normal / abnormal  3-class: normal / crackle / wheeze | a digital recording stethoscope ThinkLabs Inc. connected to a MP3 player | 44.1 KHz | Private | (16) |
| Khan, 2012 | India | Acquisition of lung sound from rural local heath worker | NA | 40 subjects (20 normal, 20 with bronchitis) | Medical center doctor | Bronchitis/ normal | Modified stethoscope connected to cell phone | 44100Hz in 16 bit PCM format | Private | (17) |
| Jin, 2014 | Singapore | NA | 11±17 years | 7 healthy / 14 pathological subjects | An experienced doctor in Singapore National University Hospital | Inspiratory/expiratory, normal/continuous adventitious sound (CAS) | ECM-77B + hemispherical rubber chamber | 11025Hz, monochannel | Private | (18) |
| Mazic, 2015 | Croatia | Children at General Hospital of Dubrovnik | 1-6 years | 16 children (45 phono-pneumograms) | NA | Wheeze/ non-wheeze | Accelerometer (BU-3173, Knowles Electronics) | 8kHz | Private | (19) |
| Milicevic, 2016 | Croatia | Children at General Hospital of Dubrovnik | 1-6 years | Wheeze/ without wheeze : 369 / 495 samples | NA | Wheeze/ non-wheeze | Transducer, preamplifier | 8kHz | Private | (20) |
| Khan, 2017 | India | Children hospitals of Pusad, Digras and Nagpur in the state of Maharashtra India | NA | 254 sounds (127 normal / 127 abnormal) | NA | Normal / adventitious lung sounds | Littman 3200 electronic stethoscope | 4kHz | Private | (21) |
| Pneumonia Etiology Research for Child Health (PERCH) study | The Gambia, Kenya, South Africa, Zambia, Bangladesh, and Thailand | World Health Organization-defined severe or very severe pneumonia / age-matched community controls | 1-59 months (median 7±11.4) | 1157 children | Nine expert reviewers (pediatricians or pediatric-experienced physicians) | Normal/ Abnormal with descriptor label: definite, probable or non-interpretable | ThinkLabs Inc. ds32a + Sony-ICD-UX71-81 | 44.1 kHz | Private | (22) |
| Mohamed, 2018 | Switzerland | Chest infection at the pediatric emergency department of the Geneva University Hospital | Under 5 years (mean 31 months) | 48 patients, 5 controls (424 recording) | NA | Bacterial pneumonia/ normal | Littman 3200 electronic stethoscope | NA | Private | (23) |
| Gouda, 2019 | Egypt | Alexandria University Children Hospital | Up to 12 years | 446 respiratory sounds | Diagnosed by a pediatrics chest consultant | Normal/wheeze/stridor | Littman 3200 electronic stethoscope | 4kHz | Private | (24) |
| Grzywalski, 2019 | Poland | Department of Paediatric Pulmonology, Karol Jonscher University Hospital in Poznan | 1-18 years (mean 8.5; median 8) | Train, test set - unknown, test set - 50 patients (522 recordings) | Five pediatricians carried out two self-reliant and independent verifications | Normal/ wheeze/ rhonchi/ coarse crackles/ fine crackles | StethoMe® and Littmann 3200 electronic stethoscopes | NA | Private | (25) |
| Liu R, 2019 | China | Pediatric clinics in Shenyang and Shanghai | Pediatric | 222 pediatric subjects (508 recordings) | NA | With/without adventitious sound | Smartho-D2 electronic stethoscope | Various | Private | (27) |
| Kotb, 2020 | Egypt | Children diagnosed with chest disease | 36.9 months (1-144) | 116 children (464 recordings | Diagnosed by clinicians | Normal/ bronchial breathing/ crepitation/ wheezes | Microphone connected to stethoscope | 44.1kHz | Private | (26) |
| Karimizadeh, 2021 | Iran | CF patients | 16.3 ± 2.7 years | 209 patients | Severity of CF classified by %FEV1 | CF severity: normal/ mild/ moderate/ severe | 37 channel microphone | 8kHz | Private | (27) |
| Kuo, 2021 | Taiwan | Kaoshiung Chang Gung Memorial Hospital (CGMH) | 0-11 years | 95 children | NA | Normal/ wheeze | Custom-built: stethoscope bell, micro-phone, and wireless signal acquisition module | 2kHz | Private | (28) |
| Cheng 2021 | Singapore | Outpatient respiratory clinic and inpatient wards at KK Women’s and Children’s Hospital, Singapore | Mean age 5.4 years (0-16) | 73 single-label samples | Two trained pediatric respiratory physicians | Normal/ crackle/ wheeze | Custom device with stethoscope head connected to a smartphone | NA | Private | (32) |
| Gelman, 2022 | Russia | Regional Children’s Clinical Hospital of Perm and the polyclinic of Perm State Medical University | 0 to 47 years (88.6% < 20-years-old) | 951 records in asthma, 167 healthy volunteers | Bronchial asthma diagnosed according to guidelines | Asthma/ healthy | Cellphone built in microphone and/or external microphone connected to cellphone or computer | NA | Private | (33) |
| Kim, 2022 | Korea | Department of Pediatrics at university hospitals in Korea | Wheezing 4 years (2-8), others 3 years (1-5) | 76 patients | Recording specialist diagnosis, validation by two pediatric pulmonologists | Wheeze/ non-wheeze | Electronic stethoscope (Jabes, GSTechnology, Seoul, Korea). | NA | Private | (34) |
| Nguyen, 2022 | USA | Children’s National Hospital’s pediatric ED with asthma exacerbation | 2-18 years | 1095 lung sound recordings | ED provider | Wheeze/ non-wheeze | StethAid | 8kHz | Private | (36) |
| DeepBreath | Switzerland, Brazil, Senegal, Cameroon, Morocco | Pediatric outpatient facilities suspected of lower respiratory tract infection, acute asthma or obstructive bronchitis | below 16 years | 572 patients, 4552 audio recordings | All diagnoses confirmed by two medical doctors. | Control/ pneumonia/ wheezing disorder/ bronchiolitis | Littman 3200 electronic stethoscope | 4kHz | Private | (41) |
| Park, 2023 | Korea | Pediatric pulmonology outpatient clinic of Seoul National University Hospital | NA | Retrospective 1022 clips, prospective 90 clips | Two pediatric pulmonology specialists | Normal/ wheezing/ crackle | ThinkLabs + Sony voice recorder | 44.1KHz | Private | (48) |
| R.A.L.E. | Canada | Dept. of Pediatrics and Child Health, University of Manitoba | All age groups | Over 50 recordings | NA | Normal/ wheezes/ crackles/ others | Contact accelerometers (EMT25C, Siemens) | 10kHz | Public | (29) |
| Chowdhury, 2024 | Bangladesh | NA | 0-5 years | 19 toddlers (72 samples) | Verified, validated and doubled-checked by medical professional. | Healthy/pneumonia | MDF-747 stethoscope + BOYA-M1-Pro electret condenser microphone | 44.1kHz | Private | (51) |
| Crisdayanti, 2024 | Korea | Pediatric patients from Woorisoa Children’s Hospital | 1-6 years | 675 patients (11,154 recordings) | Two pediatricians | Normal/ abnormal respiratory sound | NA | 4kHz | Private | (52) |
