# Supplementary material for: Artificial Intelligence Models for Pediatric Lung Sound Analysis: Systematic Review and Meta-Analysis
Source: J Med Internet Res. 2025 Apr 18;27:e66491. doi: 10.2196/66491 (PMC12048790; doi:10.2196/66491)
Supplement: Multimedia Appendix 5 [file jmir_v27i1e66491_app5.docx]

**Multimedia Appendix 5**

Quality assessment results of studies included in this review.

|  | Risk of bias | | | | Applicability | | |
| --- | --- | --- | --- | --- | --- | --- | --- |
| Author, Year | Patient selection | Index test | Reference standard | Flow and timing | Patient selection | Index test | Reference standard |
| Forkheim, 1995 | high | low | high | low | low | low | high |
| Rietveld, 1999 | high | high | high | low | low | low | low |
| Emmanouilidou, 2012 | high | high | low | ? | low | low | low |
| Khan, 2012 | high | ? | high | high | low | low | high |
| Jin, 2014 | high | high | high | low | low | low | low |
| Mazic, 2015 | high | high | ? | low | low | low | ? |
| Milicevic, 2016 | high | high | ? | low | low | low | ? |
| Khan, 2017 | high | high | high | low | low | low | high |
| Emmanouilidou. 2018 | high | low | low | low | low | low | low |
| Mohamed, 2018 | high | ? | ? | low | low | ? | high |
| Gouda, 2019 | low | low | high | low | low | low | low |
| Grzywalski, 2019 | low | high | low | low | low | high | low |
| Liu L, 2019 | high | high | ? | low | low | low | ? |
| Liu R, 2019 | high | high | ? | low | low | low | ? |
| Kotb, 2020 | high | low | high | low | low | low | high |
| Karimizadeh, 2021 | high | high | low | low | low | low | low |
| Kuo, 2021 | high | ? | ? | high | low | low | ? |
| Liu, 2021 | high | ? | ? | low | low | low | ? |
| Cheng, 2022 | low | high | low | low | low | low | low |
| Gelman, 2022 | low | low | high | low | low | low | high |
| Kim, 2022 | low | high | low | high | low | high | low |
| Ma, 2022 | low | low | low | low | low | low | low |
| Nguyen, 2022 | low | low | high | low | low | low | high |
| Zhang Q, 2022 | low | low | low | low | low | low | low |
| Li, 2022 | low | low | low | low | low | low | low |
| Zhang L,2022 | low | low | low | low | low | low | low |
| Babu, 2022 | low | low | low | low | low | low | low |
| Heitman, 2023 | low | low | low | low | low | low | high |
| Hu, 2023 | low | low | low | low | low | low | low |
| Huang, 2023-1 | high | low | low | high | low | high | high |
| Huang, 2023-2 | high | low | low | high | low | high | high |
| Ngo, 2023-1 | low | low | low | low | low | low | low |
| Ngo, 2023-2 | low | low | low | low | low | low | low |
| Ntalampiras, 2023 | low | low | low | low | low | low | low |
| Park, 2023 | high | low | low | high | low | low | low |
| Pessoa, 2023 | low | low | low | low | low | low | low |
| TaghiBeyglou, 2023 | low | high | low | high | low | low | low |
| Chowdhury, 2024 | high | high | low | low | low | low | low |
| Crisdayanti, 2024 | high | high | low | low | low | low | low |
| Wang, 2024 | low | low | low | low | low | low | low |
| Yeh, 2024 | low | high | high | high | low | high | high |
